# Effect of Decompression on Jaw Cystic Lesions Based on Three-Dimensional Volumetric Analysis

**DOI:** 10.3390/medicina56110602

**Published:** 2020-11-10

**Authors:** Yeh-Jin Kwon, Kyeong-Soo Ko, Byung-Kyu So, Dong-Hyuck Kim, Hyon-Seok Jang, Soo-Ho Kim, Eui-Seok Lee, Ho-Kyung Lim

**Affiliations:** 1 Department of Oral and Maxillofacial Surgery, Korea University Guro Hospital, Seoul 08308, Korea; yehjin51@nate.com (Y.-J.K.); k1015ks@naver.com (K.-S.K.); sobk4728@naver.com (B.-K.S.); hyperlion@naver.com (D.-H.K.); 2 Department of Oral and Maxillofacial Surgery, Korea University Ansan Hospital, Gyeonggi-do 15355, Korea; omfs1109@korea.ac.kr; 3 Department of Oral and Maxillofacial Surgery, Chungnam National University Hospital, Daejeon 35015, Korea; patron12@naver.com

**Keywords:** surgical decompression, cyst, 3D imaging, volume

## Abstract

*Background and objectives*: This study aimed to evaluate the effectiveness of decompression on jaw cysts according to various parameters by volumetric analysis using three-dimensional computed tomography. *Materials and methods*: Fifty patients who underwent surgical decompression of the jaw cystic lesion were selected, and their preoperative and postoperative computed tomography results between 3 and 27 months were collected. Volumetric analysis was performed to evaluate any differences in the rate of volumetric change according to the sex, age, initial volume of the lesion, duration, location of the lesion, tooth extraction, expansion of the cortical layer, and pathological diagnosis. Multiple linear regression and generalised linear mixed models were used for statistical analyses. *Results*: The mean reduction rate among all patients was 54.68%. Multiple linear regression analysis revealed that higher reduction rates were associated with a long decompression period, young patient age, and location of the cyst in the posterior maxilla. Generalised linear mixed models revealed that higher reduction rates were associated with a long decompression period and young patient age. *Conclusions*: Decompression was an effective treatment for reducing the cyst size in all patients. Its effectiveness increased with a long treatment duration, young patient age, and cyst location in the posterior maxilla three-dimensionally.

## 1. Introduction

Cystic lesions are one of the most common pathologies in the oral and maxillofacial area. These lesions are more frequently observed in the upper and lower jaws than other bones in the human body because a cell remnant of the embryonic neuroectoderm can trigger pathogenesis [1,2]. Standard treatment methods for cystic lesions are enucleation, which usually removes cystic walls at once, marsupialisation, which connects to the surrounding oral mucous membranes, and decompression, which seeks to form a bone by relieving pressure in the lesion [1,2]. To determine the management methods of cystic lesions, surgeons consider the size, location, pathological diagnosis, and anatomical structures of the lesions. If the lesion invades the adjacent structures, or if the primary enucleation could induce the pathological fracture or neurological damage, marsupialisation or decompression is initially considered [3].

Decompression is a technique that reduces the pressure of the cystic fluid in the cystic cavity and induces bone deposition towards the cystic wall [3,4]. If the size of the lesion is decreased after the decompression surgery and the condition of the lesion does not affect the adjacent important anatomical structures such as the inferior alveolar nerve and teeth around the lesion, the timing of cyst enucleation is determined based on the surgeons’ judgment [5,6]. In previous studies, the factors affecting cyst volume reduction were controversial, and the methods of analysis of volumetric changes varied. Moreover, several reports regarding the volumetric analysis of cysts used panoramic radiography with morphological distortion [7,8]. To overcome some of the limitations of panoramic radiography, we have tried to use computed tomography (CT) analysis to determine the three-dimensional (3D) volumetric changes in this study.

This study aimed to investigate the efficacy of decompression and analyse the factors affecting the volumetric change of cystic lesions based on sex, age, total duration of decompression, initial volume of the lesion, location of the lesion, presence of involved teeth, cortical bone expansion, and specific pathological diagnosis.

## 2. Materials and Methods

### 2.1. Patients’ Criteria

The study was conducted retrospectively on patients who underwent cystic decompression surgery in their jaws at the Department of Oral and Maxillofacial Surgery of Korea University Guro Hospital from 2012 to 2020. The inclusion criteria were as follows: patients who were diagnosed with cyst or cystic benign tumour histopathologically, patients who underwent decompression surgery based on surgeons’ decision, and patients who underwent preoperative and postoperative CT at least once. The exclusion criteria were as follows: patients who did not visit the hospital after undergoing decompression surgery, patients with omitted CT data, patients with a vanished drain during the follow-up periods, and patients having other accompanying pathologies such as sinusitis, which could affect volumetric changes, and osteoporosis, which could decrease the bone deposition ability. A total of 50 patients met the inclusion criteria and were subsequently included in our study. This study was approved by the Korea University Guro Hospital Ethics Committee (Approval No: 2020GR0450, Approval date: 7 October 2020). In addition, the clinical trial was in accordance with certification for ISO14155.

### 2.2. Surgical Procedures for Decompression

Surgical procedures of all patients were performed under the standardised protocol as follows. Decompression was performed under local anaesthesia with lidocaine, including 1:100,000 epinephrine (Yuhan Corporation, Seoul, Korea) around the lesion, accompanied by minimal incision using a #15 scalpel. If the cortical bone of the incision area was intact, an access hole was formed using high-speed handpiece and round bur. The hole size was 1 mm larger than the diameter of the tube. After approaching the cyst, a part of the cystic membrane tissue was incised and fixed with formalin solution for the histological confirmation. After inserting the Penrose drain into the cyst, the drain was sutured on the oral mucosa using the 4-0 Ethilon nylon (Ethicon, Georgia, MI, USA). The drain was cut 0.5 mm from the mucosa. After suturing, irrigation was performed several times with normal saline using a tube. After the operation, all patients were prescribed cefaclor hydrate 250 mg (thrice a day), aceclofenac 100 mg (twice a day), and esomeprazole 20 mg orally (once a day), and a 0.2% chlorhexidine mouthwash was topically rinsed (for 1 min, thrice a day) for 7 days. For patients with allergy or taking other drugs, replaced prescription was made in consideration of its interaction with the other drugs. If a third molar or another tooth was involved with the cyst, extraction was initially performed, and the insertion of a tube was performed consecutively. Patients were trained to irrigate themselves with sterile saline. After the decompression surgery, the surgeon assessed the drain condition monthly. If the drain was contaminated, it was replaced with a new one.

### 2.3. Computed Tomography Data Acquisition

3D CT data using the cone beam CT device (Vatech, Seoul, Korea) or medical CT device (Siemens Definition Edge, Erlangen, Germany) were obtained. A cut of at least a 1-mm slice was obtained from all CTs. All patients underwent CT before surgery and once or several times after surgery between 3 and 27 months.

### 2.4. 3D Volume Measurements

**Figure 1:**
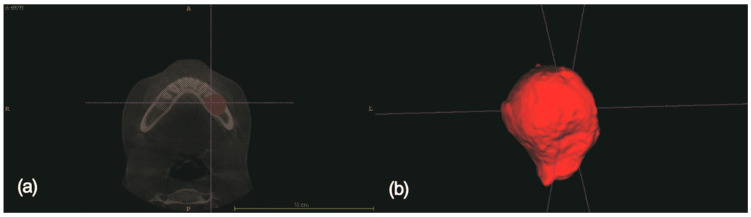
(**a**) Segmentation of the cystic lesion in axial plane of computed tomographic data. (**b**) Computerized three-dimensional reconstruction of the cystic lesion. Volume was measured with ITK-SNAP (Penn Image Computing and Science Laboratory, PA, USA).

The open-source software ITK-SNAP (Penn Image Computing and Science Laboratory, PA, USA) was used to measure the volume of the cystic lesion. The CT digital imaging and communications in medicine (DICOM) datasets were imported into the program and were sliced in the axial plane. In setting the cystic lesion margin, Hounsfield unit (HU) values of the 3D voxel were designated in slice. We assumed that the cystic cavity had the value of the lower threshold range from −1000 to −500 HU and the upper threshold range from 500 to 1000 HU in CT slice. When the surface boundary of the intra-bony cyst bone was clear, semi-automatic segmentation, a preprogrammed automatic system was performed. However, when the surface boundary line was unclear, manual segmentation was performed. After the designation of the cystic area in each slice, a 3D volumetric image was rendered by the sum of each slice in axial plane (Figure 1a), and 3D cystic volume was measured and calculated (Figure 1b). In cases involving teeth, to remove the volume of teeth, the volume of extraction socket was excluded in the total volume. Comparing between preoperative and postoperative volume measurements, the percentage reduction in the volume (PRV) of cystic lesions in the jaw was calculated as follows:

(1) $$\mathrm{PRV}\left(\%\right)=\;\frac{(\mathrm{Cystic}\;\mathrm{Volume}\;\mathrm{before}\;\mathrm{Decompression}-\mathrm{Cystic}\;\mathrm{Volume}\;\mathrm{after}\;\mathrm{Decompression})}{\mathrm{Cystic}\;\mathrm{Volume}\;\mathrm{before}\;\mathrm{Decompression}}\;\times 100$$

### 2.5. Determination of Effective Parameters

We assumed that the following parameters could influence the effect of decompression: sex, age, total duration of decompression, initial volume of the lesion, location of the lesion, presence of involved teeth, cortical bone expansion, and a specific pathological diagnosis.

Considering the low resistance to expansion pressure of the maxillary sinus, it was classified into three areas based on the anatomical location (anterior maxilla, posterior maxilla, and mandible). Considering the degree of cortical layer expansion, the criterion was also based on the results of the study by Jeong et al. [3]. Cortical layers expanding greater than 1.5 times buccolingually than the opposite normal jaw bone were classified as severe, while those expanding less than 1.5 times were classified as mild. Based on the pathological diagnosis, cysts were divided into the following three groups: dentigerous cyst, radicular cyst, and odontogenic keratocyst. The clinical information from the enrolled patients was investigated.

### 2.6. Statistical Analyses

To determine the factors affecting PRV, two statistical analysis methods were used in this study. First, to correct the disturbance among factors influencing each other, multiple linear regression analysis was performed with only two images: the pre-decompression image and the final image acquired just before enucleation. Second, to correct the disturbance in factors caused by irregular CT scan timings, a generalised linear mixed model was used, including all images acquired during the complete follow-up process. All statistical analyses were performed with Statistical Analysis System (SAS) version 9.4 (SAS Institute, Cary, NC, USA). The significance level was set to *p* < 0.05.

## 3. Results

### 3.1. Patient Distribution

Patients were classified according to sex, age, initial volume of the lesion, duration, location of the lesion, presence of involved teeth, cortical bone expansion, and pathological diagnosis (Table 1). The total number of samples was 50 (37 male and 13 female), with an average age of 32.3 years (range, 7–71 years). The average initial cyst volume was 7.27 mL (range, 0.77–36.65 mL). The mean duration of decompression was 9.41 months (range, 3–27 months). The cystic lesions were located in the anterior maxilla, posterior maxilla, and mandible in 14, 7, and 29 patients, respectively. The number of cases involving the teeth was 12. The numbers of patients with mild and severe cortical expansions were 16 and 34, respectively. The pathological diagnoses were as follows: 17 dentigerous cysts, 26 radicular cysts, and 7 odontogenic keratocysts (Table 1).

All patients showed a reduction in the cyst volume. The average decompression period was 9.41 months. The duration for decompression varied from at least 3 months to a maximum of 27 months, and the value of PRV varied from up to 77.6% to at least 33.9%.

### 3.2. Effective Factors in the Two Statistical Analyses

**Figure 2:**
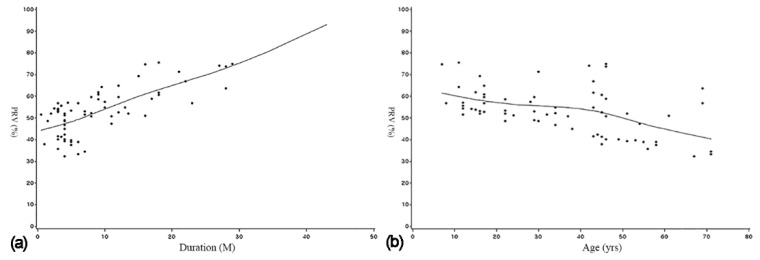
(**a**) Regression graph between the independent variable duration and the dependent variable PRV. (**b**) Regression graph between the independent variable age and the dependent variable PRV.

The linear multiple regression analysis revealed significant differences in reduction rates with the patient age and duration and location of the lesion. Higher reduction rates were found with the young patient age (*p* = 0.037), long duration of decompression (*p* < 0.0001), and cyst location in the posterior maxilla (*p* = 0.0081). Generalised linear mixed models revealed significant differences in reduction rates with the patient age and duration of the lesion. Higher reduction rates were found with the young patient age (*p* = 0.0124) and long duration of decompression (*p* = 0.0016; Figure 2; Table 2). The regression equation derived from the two analysis methods is as follows:

(2) y=56.3067 + 1.3396 × duration−0.4376 × age

There was no statistically significant difference in any other factor (sex, initial cystic volume, involved teeth, cortical bone expansion, or pathological diagnosis).

## 4. Discussion

Since cystic decompression was introduced in the conservative treatment of odontogenic cysts, several cases of treatment with decompression have been encountered, and a high success rate has been reported in various studies [9,10,11]. Because decompression is a long-term treatment, it is important to determine the optimal timing for cyst enucleation [3]. There are no official criteria for decompression periods or size changes [11]. After the proper removal of a lesion, a surgeon would decide on the optimal enucleation timing [5,6]. Therefore, PRV investigation of the cystic lesion is beneficial to achieve clinical success.

In previous studies, there was no disagreement about the effectiveness of volume reduction after cystic decompression. However, there are various opinions about the ideal period of cystic decompression. Marker et al. reported that it is desirable to perform cyst enucleation if the size of the cystic lesion after decompression has shrunk greater than 50%–60% [9]. In general, 6 to 14 months was suggested as the appropriate period of cystic decompression in most reported studies [9,10,12,13,14,15,16,17,18]. However, Anavi et al. reported that a long period of cystic decompression of more than 33 months in the maxilla and 22 months in the mandible before cyst enucleation was ideal [13]. Yi Zhao et al. suggested that the cystic volume would decrease significantly within 3 months after decompression surgery [12], and Bodner et al. reported that a minimum decompression period is required for safe cyst enucleation [19]. In summary, previous reports suggest a period of 3–14 months and a period of 50–60% reduction in size as the ideal period of decompression. The mean PRV was 54.68%, and the mean period of decompression was 9.41 months (range, 3–27 months) in our study, which was similar to the results of the other studies.

Comparing volumetric changes in cystic lesions before and after decompression has been attempted with several analysis methods. Yi Zhao et al. obtained the cyst volume by injecting physiological saline into an empty cystic cavity and calculating the volume of the injected liquid [12]. In most studies, preoperative and postoperative panoramic radiographs or CT images were used [2,5,13,14,17,20,21,22]. In early days, studies utilized panoramic radiography when assessing the volumetric changes in the jaw cysts after decompression surgery [2,13]. With panoramic radiography, patients are less exposed to radiation, and it is more cost-effective than CTs. However, due to the limitation of the two-dimensional analysis of panoramic radiography, it was difficult to determine the exact volumetric changes [3]. Moreover, it is difficult to determine the exact three-dimensional association between the adjacent anatomical structures and lesions. Therefore, recently, several studies assessing the more accurate volumetric changes using 3D-CT data were been conducted [1,3,23]. Using 3D-CT, a relatively accurate shape of cysts can be determined by considering the variable shapes of the lesions, and accurate determination of volumetric changes after decompression is possible [1,3]. Furthermore, in our study, using the concept of the integral, a more precise and clearer study could be conducted using 3D-CT and a software.

The authors have previously suggested factors that affect graft failure during enucleation [24]. Graft failure with concomitant cyst enucleation could increase in cases of younger age, smoking, preoperative infection, large size, impaction of the mandibular third molar, perilesional osteosclerosis, and the use of mixed non-autogenous and autogenous bone [24]. In this study on decompression, it was confirmed that cases of long period, in younger patients, and in the posterior maxilla showed more volume reduction effect.

After the parameters had been evaluated in terms of its effect on decompression, age was considered statistically significant (*p* < 0.05). The effects of decompression based on age are controversial. Although one study reported that younger patients had a higher PRV [13], another study reported that decompression is not associated with age [25].

Longer cystic decompression periods were associated with significantly greater effectiveness of decompression (*p* < 0.05) in our study. Most previous studies were designed without considering the cystic reduction velocity during decompression, whereas some were designed considering cystic decompression velocity. Some studies showed that the volume reduction rate was higher during the initial decompression period, implying that the rate of reduction varies from with time during the period of cystic decompression [12,20].

The mean PRVs were 67.3%, 53.9%, and 52.0% in the posterior maxilla, anterior maxilla, and mandible, respectively, in our study, showing statistically significant differences (*p* < 0.05). The outcome of decompression revealed that the PRV was more favourable in the posterior maxilla, with maxillary sinus involvement, than the anterior maxilla or mandible. Jeong et al. reported that the PRV in the maxilla is significantly favourable because of the maxillary sinus, which contains several air spaces [3].

In our study, initial cystic volume was considered insignificant. Jeong et al. reported that the rate of reduction of volume was associated with the initial size of the lesion—specifically, with the group with large initial volume lesions [3]. Song et al. [21] also reported that the rate of reduction of volume was associated with the original size of the lesion [1]. However, Anavi et al. reported that the initial size did not affect the outcome of decompression [13].

The effects of the other parameters were unclear. In this study, there were no statistically significant differences in terms of tooth extraction and the rate of expansion of the cortical layer. These results were consistent to the results of the study conducted by Jeong et al. [3]. Pathological diagnosis did not influence the effect of decompression in our study, as also reported in the study by Anavi et al. [13]. However, in several studies, decompression of OKC showed more favourable results than in other types of cysts [7,26,27].

Considering that this study had a retrospective study design, the timing of CT scans and follow-up duration were inconsistent. Although we found that decompression was associated with young age, duration, and location of lesion, considering the limitation of this study, more accurate and precise clinical guidelines are required. Selective application of decompression surgery in jaw cystic lesions is a good choice to achieve an optimal therapeutic effect.

## 5. Conclusions

The conclusions of this study are as follows.

1. In the patient group to which the decompression procedure was applied, an average of 54.68% reduction in cyst size was observed during an average observation period of 9.41 months.
2. Decompression is an effective procedure for reducing the size of cysts in all patients, as determined by 3D volumetric analysis.
3. Decompression was more effective when the procedure was applied for a long period, in younger patients, and in the posterior maxilla.

## Figures and Tables

**Table 1:** Percentage reduction in volume of 50 cysts according to parameters *.

Parameter		Number	PRV (%)	
			Mean	SD
Sex	Male	37	55.812	23.72
	Female	13	51.469	29.83
Location	Mandible	29	52.006	23.95
	Anterior maxilla	14	53.943	29.52
	Posterior maxilla	7	67.254	20.05
Tooth extraction	Extraction	12	55.491	26.06
	No extraction	38	54.428	25.28
Expansion of the cortical layer	Mild	16	60.826	23.36
	Severe	34	51.792	25.85
Pathological diagnosis	DC	17	55.272	25.24
	RC	26	55.192	25.27
	OKC	7	51.362	28.52
Total		50	54.683	25.20

PRV, percentage reduction in volume; SD, standard deviation; DC, dentigerous cyst; RC, radicular cyst; OKC, odontogenic keratocyst; * Age, initial cystic volume, and duration were excluded in the table since these factors were not categorical but numerical data.

**Table 2:** Comparison of 50 cystic cases with two statistical methods.

Parameter	*p*-Value with the Multiple Linear Regression Analysis	*p*-Value with Generalised Linear Mixed Models
Sex	0.1869	0.7712
Age (years)	0.037 *	0.0124 *
Initial cystic volume (mL)	0.1661	0.1711
Duration (M)	<0.0001 *	0.0016 *
Location	0.0081 *	0.2156
Tooth extraction	0.2628	0.4438
Expansion of cortical layer	0.1036	0.4582
Pathological diagnosis	0.2803	0.894

* Significantly different mean percentage reduction in volume (*p* < 0.05).
